# Why do bacteria divide?

**DOI:** 10.3389/fmicb.2015.00322

**Published:** 2015-04-16

**Authors:** Vic Norris

**Affiliations:** Laboratory of Microbiology Signals and Microenvironment, Theoretical Biology Unit, University of Rouen, Mont Saint AignanFrance

**Keywords:** neural net, FtsZ, connectivity, heterogeneity, molecular assembly, dualism, competitive coherence, hyperstructure

## Abstract

The problem of not only how but also *why* cells divide can be tackled using recent ideas. One idea from the origins of life – *Life as independent of its constituents* – is that a living entity like a cell is a particular pattern of connectivity between its constituents. This means that if the growing cell were just to get bigger the average connectivity between its constituents per unit mass – its *cellular connectivity* – would decrease and the cell would lose its identity. The solution is division which restores connectivity. The corollary is that the cell senses decreasing cellular connectivity and uses this information to trigger division. A second idea from phenotypic diversity – *Life on the Scales of Equilibria* – is that a bacterium must find strategies that allow it to both survive and grow. This means that it has learnt to reconcile the opposing constraints that these strategies impose. The solution is that the cell cycle generates daughter cells with different phenotypes based on sufficiently complex equilibrium (**E**) and non-equilibrium (**NE**) cellular compounds and structures appropriate for survival and growth, respectively, *alias* ‘hyperstructures.’ The corollary is that the cell senses both the quantity of **E** material and the intensity of use of **NE** material and then uses this information to trigger the cell cycle. A third idea from artificial intelligence – *Competitive Coherence* – is that a cell selects the *active* subset of elements that actively determine its phenotype from a much larger set of available elements. This means that the selection of an active subset of a specific size and composition must be done so as to generate both a coherent cell state, in which the cell’s contents work together harmoniously, and a coherent sequence of cell states, each coherent with respect to itself and to an unpredictable environment. The solution is the use of a range of mechanisms ranging from hyperstructure dynamics to the cell cycle itself.

## Introduction

The very fact of growing is a source of many serious problems that bacteria somehow have to solve. These problems include DNA becoming a limiting factor (thereby disrupting patterns of expression and preventing exponential growth), positive feedback (thereby leading to a single phenotype), over-reliance on fragile, non-equilibrium (**NE**) structures such as those created by the coupling of transcription, translation (thereby making the cell vulnerable to changes in its environment), and unbalanced production of RNA, protein and lipid (Norris, 2011). The problems do not stop there. More generally, bacteria must have found ways to avoid incoherence at the level of both the individual cell and the population so as to be able to not only grow but also survive (arguably, the more important of the two behaviors since a dead cell can never grow) in what we have called ‘Life on the Scales’ (Norris and Amar, 2012a). More generally still, bacteria – like all living objects – risk losing the connectivity between their constituents as they get bigger (Norris, 2015). This is particularly serious because patterns of connectivity are often claimed to define pretty well everything – as testified by the claims of universality made for self-organized criticality (Bak, 1996) and small world networks (Watts and Strogatz, 1999) – so that a bacterium that continued to grow would eventually lose its identity.

The above problems are intimately related to the cell cycle. In non-differentiating bacteria such as *Escherichia coli*, the cell cycle was long considered to have only the function of replicating the hereditary material and distributing it into two cells that would be effectively identical unless driven down the path of differentiation. This fitted with the idea that the laws underlying cell growth – and, in particular, those directly relevant to the cell cycle – could be found not by studying single cells but rather by studying cells as an aggregate (Cooper, 2006). However, it is now clear that even *E. coli* generates substantial phenotypic diversity (Godin et al., 2010; Wang et al., 2010; Kiviet et al., 2014; Osella et al., 2014; Taheri-Araghi et al., 2015) with cell division playing the key role. It is not therefore surprising that connections have been found between the replication of the bacterial chromosome and processes that include central carbon metabolism (Janniere et al., 2007; Baranska et al., 2013), phospholipid synthesis (Sekimizu and Kornberg, 1988; Xia and Dowhan, 1995), respiration, lipoteichoic acid synthesis, and ribosome biosynthesis (Murray and Koh, 2014). Other connections have also been found between cell division and processes that include fatty acid synthesis (Yao et al., 2012), glycolysis and its offshoots (Weart et al., 2007; Hill et al., 2013; Monahan et al., 2014), and polyphosphate metabolism (Rao et al., 2009; Boutte et al., 2012).

In the above context, it is interesting to revisit cell division in terms of concepts, such as *hyperstructures* or *competitive coherence*, that are either new or under development. The concept of *hyperstructures* is directly relevant to the problem of maintaining connectivity. A hyperstructure is an assembly of elements (such as genes, RNA, proteins, small molecules, and ions) that performs a function and that constitutes a substantial proportion of the cellular material (Norris et al., 2007a; Saier, 2013; Meyer et al., 2014). The existence of the hyperstructure level is a partial solution to the problem of reducing the enormity of phenotype space from the combinations of 1000s of types of macromolecules to combinations of a 100 or so hyperstructures, thereby allowing Darwinian selection to operate (Kauffman, 1996; Norris et al., 2014). Hyperstructures exist in **NE** and equilibrium (**E**) forms with the ratio between these forms proposed to control the cell cycle and to generate phenotype diversity by sensing both the intensity with which **NE** hyperstructures are working and the quantity of **E** hyperstructures that has accumulated (Norris, 2011; Norris and Amar, 2012a); the importance of cell division to the related stability of cellular constituents has been demonstrated by simulation (Kamimura and Kaneko, 2014). The concept of *competitive coherence*, which relies on patterns of connections, has been developed to explain the behavior of biological systems, which include, of course, bacteria (Norris et al., 2014). This behavior is determined by a relatively small, phenotypically *active*, subset of all the constituents available to the bacterium; selection of this subset entails competition between (1) those constituents whose activities are coherent with one another and with the environment and (2) those constituents whose activities are coherent with the previous history of the bacterium. Simulation has shown that a system based on competitive coherence can adapt to growth and stress conditions (Norris et al., 2012). Moreover, the concept can be extended to include the role of **NE** and **E** hyperstructures and the role of the cell cycle in the nature and size of the active subset (Norris et al., 2014).

According to Krakauer (2014) at the Santa Fe Institute, this institute “is about circling a phenomenon, considering all the angles and unique perspectives to see a thing in a completely new light." Such circling could be likened to someone preparing to climb a mountain shrouded in fog who tries many routes that may be useful-even if they do not lead to the summit-because they give new views. This is the approach to cell division that I adopt here.

## Maintenance of Connectivity

**FIGURE 1:**
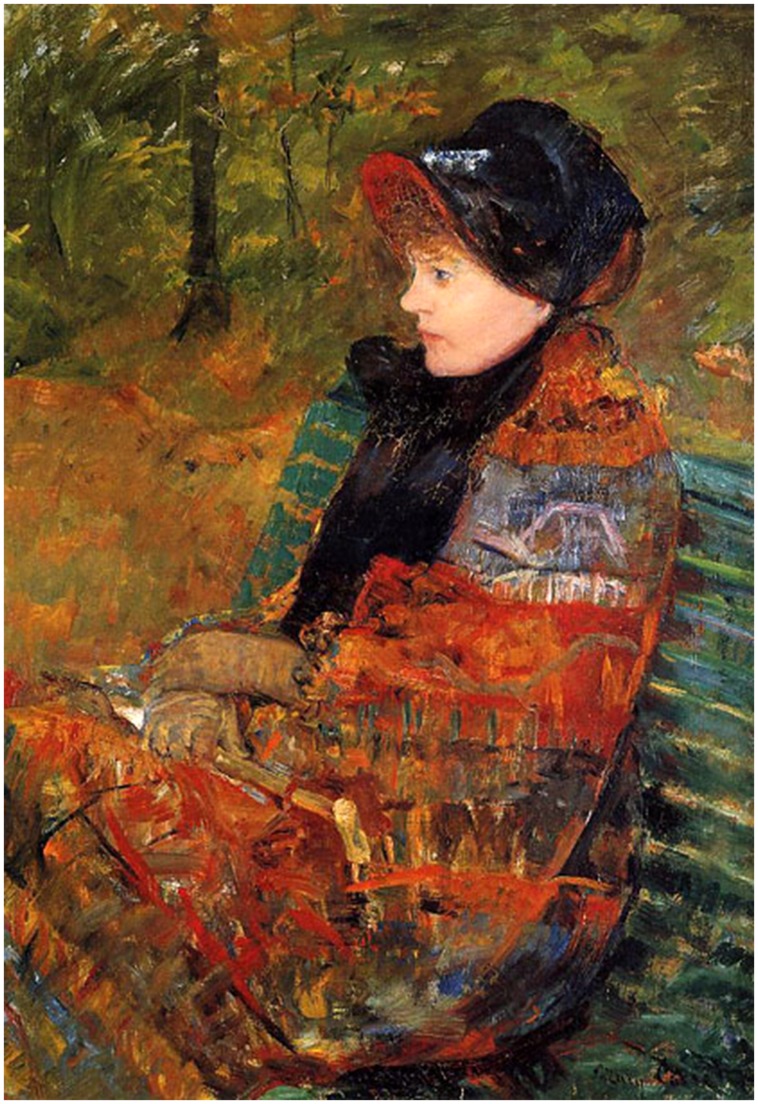
**Profile of Lydia Cassatt (1880)**. This portrait entitled Autumn of the seriously ill Lydia by her sister, Mary Cassatt, can be interpreted as showing her losing her identity (copied from http://hoocher.com/Mary_Cassatt/Mary_Cassatt.htm original in the Musee du Petit Palais, Paris, France).

A bacterium is a physical object but what exactly is a physical object? Put differently, what makes one physical object different from another. A crab-like alien examining the Earth from orbit might mistake me and the chair on which I am sitting for an eight-legged crab. The reason the alien would be wrong is that there is a frontier between my chair and me. My constituent bits or elements are more connected to one another than they are to the chair’s bits and *vice versa*. More generally, a physical object can be said to exist in some space if there is a topologically closed (or nearly closed) discontinuity in the connectivity of the elements in that space; this discontinuity constitutes a frontier of connectivity that makes the object different from its environment. The exact nature of this object is defined by the pattern of connectivity of its constituent elements and by the pattern of connectivity of these elements to its environment. In other words, a physical object owes its identity and its very existence to patterns of connectivity. This is one interpretation that can be made of the picture by Mary Cassatt of her dying sister, Lydia (**Figure 1**).

**FIGURE 2:**
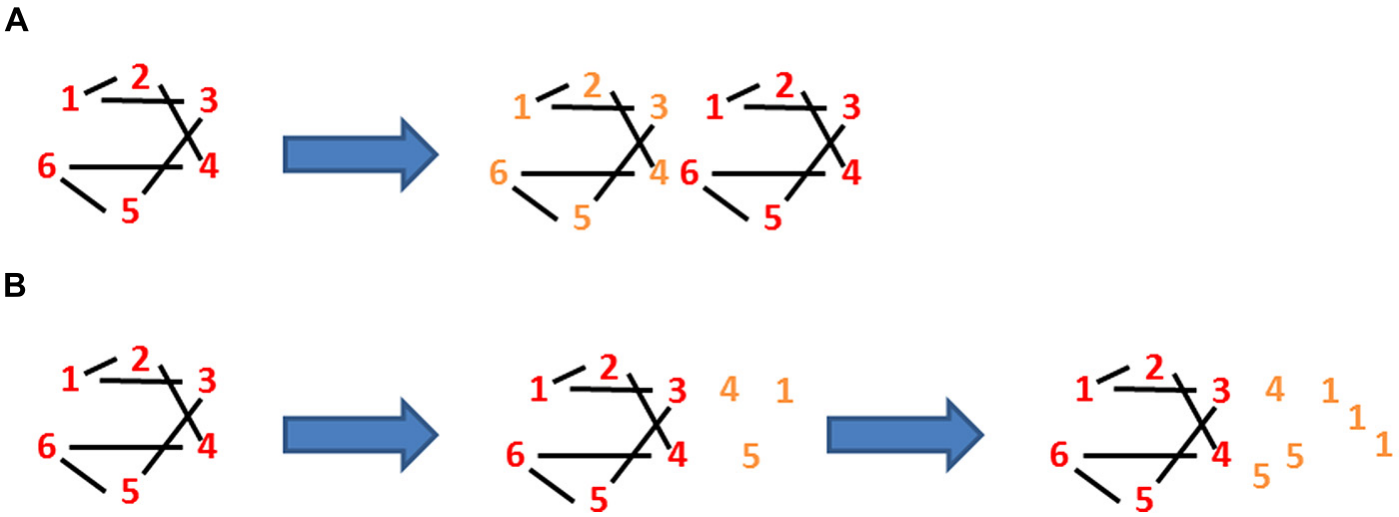
**Loss of identity through growth**. There are six elements in the autocatalytic set linked by two links per element. **(A)** The links:elements ratio (L:E) is 1 and stays 1 in the above case as the cell grows. However, this ratio per mass goes down with growth as (L:E)/M goes to (L:E)/2M. **(B)** After the first stage of growth (50% increase in mass), the (L:E)/M has gone down from 1 to (6:9)/1.5M, i.e., 0.4444 whilst after continuing but unbalanced growth (100% increase in mass), the (L:E)/M has gone down from 1 to (6:12)/2M, i.e., 0.25.

A bacterium is, of course, more than just a physical object, it is a *living* physical object. One of the characteristics of living objects such as bacteria is that at some stage they grow (or, at least, their ancestors grew). Growth entails an increase in mass *via* the addition of new elements or constituents. In the case of a bacterium, these constituents may be the same as those already there (as shown for the constituents numbered 1–6 in **Figure 2A**); hence, the addition of these new constituents may leave the average connectivity unchanged, where connectivity, L/E, equals the number of links or physical associations, L, divided by the number of constituents or elements, **E** (**Figure 2A**). In other words, growth does not necessarily change the number or nature of the connections between the constituents themselves and L/E either remains constant (**Figure 2A**) or even drops (**Figure 2B**). Growth does, of course, mean an increase in the mass of the bacterium and this in turn means that the average connectivity *per mass M of the bacterium*, (L/E)/M *alias cellular connectivity*, decreases. To make this clearer, consider a toy cell that comprises six different elements each of which binds to two other elements (such that the connectivity, L/E, is one). Consider too that this cell manages to grow so as to duplicate exactly its six constituents, as shown by the two sets of differently colored constituents in **Figure 2A**. Its L/E either remains one or, if this duplication is not exact, drops below one, as shown by the relative excess of constituents number 1 in **Figure 2B**. However, connectivity divided by mass has halved. Why does this matter?

Scaling up a process or a structure often results in a changed connectivity and a changed behavior. This is one of the dangers posed by growth: identity is changed. Consider a bacterium that has grown much bigger than normal. It would have different mechanical properties (and different resistance to mechanical stresses), a different motility, differences in regulatory networks (the behavior of the ensemble of two identical networks can be qualitatively different from that of one of these networks on its own), possibly differences in the surface/volume ratio (see, Bendezu and de Boer, 2008), etc. Not surprisingly, the changing connections that accompany increased hydration create problems for cells (Lang, 2011). The change in identity that accompanies extensive growth is not always negative. Such growth is, for example, fundamental to the change in the identity of an *E. coli* cell as it becomes a filamentous, multinucleate, hyperflagellate cell that can swarm over the surface of solid media (Harshey and Matsuyama, 1994). That said, the reduction of cellular connectivity that accompanies growth generally constitutes a serious problem constraining the evolution of cells.

It turns out that there are several possible solutions to the problem of how to maintain cellular connectivity despite growth. One of them involves the creation or the expansion of an additional level of organization. Multi-level structuring is a fundamental characteristic of living systems. Each new emergent level of organization in the dynamics of a complex self-organizing system – such as a living system – re-organizes variety on the level below so that it has meaning for the level above (Salthe, 1985; Lemke, 2000). The physical nature of the processes, the spatio-temporal scales, and connections at a particular level may be characteristic of that level (Norris et al., 2007a). In the case of the individual bacterium, I would argue that the level of organization that is relevant to the cellular connectivity problem is that of hyperstructures, which is the level intermediate between macromolecules and the bacterial cell itself (Norris et al., 2007a). There are several types of hyperstructure (Norris et al., 2007a). In the case of enzymic hyperstructures, a recent study suggests that a quarter of known cytoplasmic enzymes are in hyperstructures in which they have a higher within-group connectivity than enzymes that appear to be outside hyperstructures (Meyer et al., 2014). The relevance of the hyperstructure concept to growth is that, as the bacterium grows, the dynamics of hyperstructures changes such that some hyperstructures are born and others are consolidated; in other words, connectivity at the level of hyperstructures increases, thereby compensating for the decrease in connectivity at the level of macromolecules and maintaining the average connectivity per mass (i.e., cellular connectivity). A prime candidate for a hyperstructure that is born during growth would be the initiation hyperstructure based on the DnaA protein whilst candidates for hyperstructures that are consolidated during growth would include the ribosomal hyperstructure or microcompartment where ribosomal constituents are made and perhaps assembled (see, Intensity Sensing and Quantity Sensing; Norris and Amar, 2012a). These different hyperstructures and their dynamics may be in part responsible for the relationship between the fluidity of the cytoplasm and metabolism (Parry et al., 2014).

Another possible solution to the cellular connectivity problem involves cell division. To simplify it, cell division produces two daughter cells in which the average connectivity per constituent per mass is restored to that of the parental cell when it was born. A population of bacteria such as *E. coli* usually has a size distribution – characteristic of the rate of growth – that is the culmination of the cell cycle in which mass plays an important regulatory role (see below; Schaechter et al., 1958). It has been proposed that the timing and locating of the division site are the result of what is essentially a local reduction in the connectivity of the membrane *alias* the frontier of discontinuity with the environment (Norris et al., 2004). Evidence for this includes the appearance of membrane domains at the site of division (Mileykovskaya and Dowhan, 2000; Kawai et al., 2004), which constitutes the earliest known step of the formation of the division site in *E. coli* (Fishov and Woldringh, 1999). These anionic lipid-rich domains form in the regions outside (including between) the chromosomes, presumably by default because the membrane around the chromosomes is both enriched in other lipids and structured by the coupling between the processes of transcription, translation, and insertion of nascent proteins into membrane *alias transertion* which increases the microviscosity of the membrane (Binenbaum et al., 1999). Such relatively unconnected domains at the division site may have a propensity to invaginate as shown in phase separation experiments (Li et al., 2011). Moreover, excess production of membrane – which I would argue results in a region of relatively unconnected membrane – results in division in wall-less bacteria (Mercier et al., 2013) and can titrate division enzymes such as FtsZ and MinD in certain shape mutants of *E. coli* (Bendezu and de Boer, 2008). A lower density of connections between envelope proteins, peptidoglycan, and the inner membrane has been proposed to explain the release of membrane vesicles from Gram-negative bacteria and, in particular, from their the division sites (Deatherage et al., 2009). Despite my focus here on the membrane as the important frontier of connectivity, it should be noted that two classes of models of the cell are proposed to exist, one in which the membrane is all-important in defining the cell and a second in which the entire cell is in a different phase relative to its surroundings (Jaeken and Vasilievich Matveev, 2012). Hence, a division site might result not only from a local reduction in the connectivity of the membrane but also from a frontier of discontinuity forming in the interior of the cell (as might be argued in the case of the phragmoplast in plant cells). Indeed, when two liquid phases are in contact with a membrane, their interactions with that membrane can lead to budding (Li et al., 2012).

## Dualism

An apparently intractable dilemma often confronts bacteria: either to interact, take risks and grow, or to minimize interactions, avoid risks, shut down, and survive. A bacterium that tries to grow forever will eventually run out of resources and to survive must abandon this strategy. Conversely, a bacterium that refuses to grow whilst others grow successfully must abandon this strategy if it is not to be out-competed. A strong evolutionary pressure therefore exists to compel bacteria to choose between at least two apparently incompatible strategies or, to put it differently, to navigate in phenotype space between the two basins of growth and survival. This is the problem of *Life on the Scales* (Norris and Amar, 2012b).

The solution adopted by bacteria is to generate a phenotypically diverse population (Smits et al., 2006; Maisonneuve and Gerdes, 2014; Serra et al., 2014). This diversity extends to the growth rate itself such that, within a growing population of *E. coli*, non-growing persisters are produced at a low frequency (Balaban et al., 2004). Growth rate diversity is, however, much greater than this. Measurements of the buoyant mass of individual *E. coli* reveal a considerable variation in ‘instantaneous’ growth rates (Godin et al., 2010) as do measurements of the elongation rates of individual *E. coli* (Wang et al., 2010; Kiviet et al., 2014; Osella et al., 2014).

The many ways to achieve a phenotypically diverse population include multi-stationarity in networks (Thomas, 1980; Dubnau and Losick, 2006), noise (Silva-Rocha and de Lorenzo, 2010), spontaneous gene amplification (Anderson and Roth, 1977), and chemical communication (Vega et al., 2012). These mechanisms may not necessarily lead to the satisfaction of two requirements: the phenotypes generated need to be both (1) internally coherent and (2) coherent with respect to the other phenotypes in the population. In the case of the former requirement, if an evolutionarily useful, phenotypic diversity is to be generated, each phenotype must be coherent with respect to the set of genes expressed (Norris and Amar, 2012a). This requirement for coherence is strong in the case of the growth rate since growth and survival strategies are incompatible at the level of the individual cell.

An evident mechanism to generate coherent phenotypic diversity is the cell cycle itself. Each division in differentiating, bacterial species such as *Caulobacter crescentus* generates two cells with very different metabolisms (Bowman et al., 2013). It is therefore conceivable that even in non-differentiating bacteria such as *E. coli*, the cell cycle is the primary mechanism for generating daughter cells with different growth rates. Indeed, the authors’ interpretation of the results of a microfluidics-based experiment on the growth rate distribution of *E. coli* is that the cell “forgets" on division its growth rate in the previous cell cycle (Wang et al., 2010); further analysis of these results confirms the importance of cell division in generating diversity (Osella et al., 2014). The question is how.

The answer is that differentiation far from being difficult to achieve is difficult to avoid. Differentiation is almost inescapable in systems where negative feedback operates globally and positive feedback operates locally (Norris, 1995b; Norris and Madsen, 1995). Consider two similar research laboratories competing with others for limited funds (i.e., a globally negative feedback); if one of these laboratories is actually funded and the other is not, in the next competition, the previously funded laboratory is at an advantage (i.e., a locally positive feedback). During the cell cycle of modern bacteria, two – what are for my purpose here – essentially identical chromosomes compete for limiting factors like RNA polymerases and ribosomes; this constitutes negative regulation *in trans*. At the same time, a region of one chromosome that is being expressed by polymerases is pulled by these polymerases out of the nucleoid (where it would otherwise be buried and relatively inaccessible) and this expression increases the region’s accessibility to more polymerases; this constitutes positive regulation *in cis*; in addition, transertion allows ribosomal subunits to penetrate the nucleoid and to perform co-transcriptional translation (Bakshi et al., 2014), which in turn would protect nascent mRNA (Deana and Belasco, 2005; Deneke et al., 2013), limit the formation of the RNA degradosome (Strahl et al., 2015) and stop RNA polymerases backtracking (McGary and Nudler, 2013). The two chromosomes therefore spontaneously have different patterns of expression (Norris, 1995b; Norris and Madsen, 1995). This ‘globally negative, locally positive’ mechanism might explain the generation of daughters with different phenotypes but not the coherence of these phenotypes.

**FIGURE 3:**
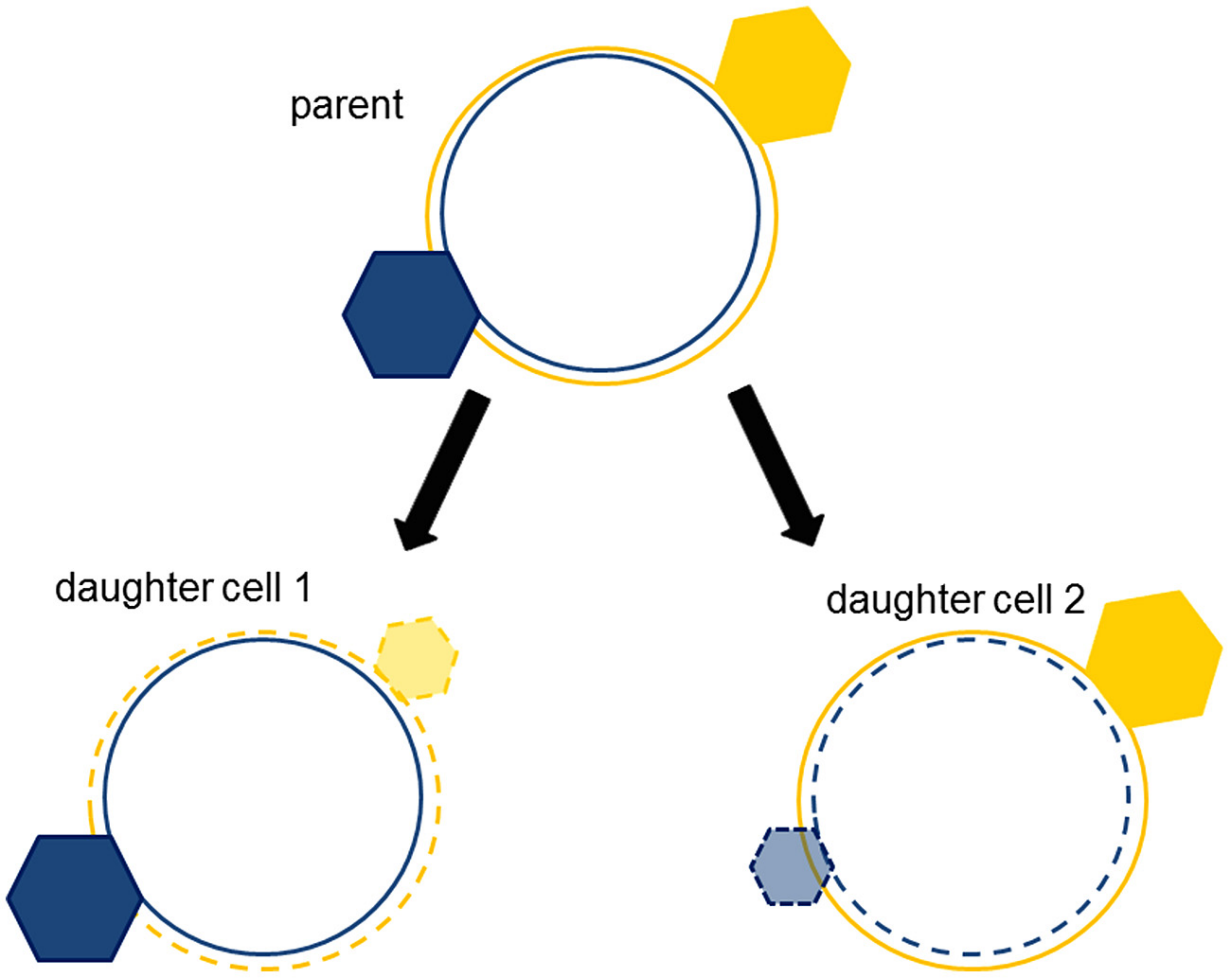
**Strand segregation of hyperstructures creates complementary, coherent phenotypes**. The hyperstructure (hexagon) associated with an old strand (continuous circle) is segregated with the old strand into the daughter cell. New hyperstructures (translucent hexagons) associated with the new strands (dotted circles) are smaller because they must compete with the parent cell’s hyperstructures (solid hexagons).

There are many ways to achieve a diversity that is coherent (Norris et al., 2014). One of them is *dualism* (Norris, 2011). It works like this: the cell cycle generates two daughter cells with different ratios of **NE** and **E** hyperstructures; these different ratios confer different properties on the daughter cells such that those with higher **NE**:**E** ratios grow fast to profit from nutrient availability whilst those with lower **NE**:**E** ratios grow slowly or cease growth so as to resist stresses. These ratios give rise to coherent phenotypes because one of the parental strands of DNA is physically associated with the **NE** hyperstructures appropriate for growth whilst the other strand is physically associated with the **E** hyperstructures appropriate for survival (Rocha et al., 2003). The logic here is that, in a competitive cytoplasm, an old strand associated with a particular hyperstructure (e.g., *via* coupled transcription–translation) has more chance of retaining that hyperstructure than a chemically identical, newly replicated (and therefore unexpressed) strand has of acquiring that hyperstructure (**Figure 3**). Hence, the older strands have the advantage in terms of making the major contribution to the phenotypes of the daughter cells. Evidence consistent with dualism includes the different locations of the leading and lagging strands (White et al., 2008) and the colocation of genes and their products (Llopis et al., 2010). In the dualism hypothesis, it is essential to achieving a phenotypically diverse population that the replication of the chromosome be followed by cell division since this puts these chromosomes into separate cells. Coherent separation leading to division can, of course, be achieved by several, complementary, physical mechanisms as shown by lipid vesicles that encapsulate an aqueous two-phase system of polyethylene glycol and dextran within a membrane of liquid-disordered and liquid-ordered domains (Andes-Koback and Keating, 2011). The philosophical rationale behind this physical separation is that the regulatory and metabolic networks that depend on these chromosomes can disrupt one another if they are not separated, as illustrated by a simple model based on artificial chemistry (Demarty et al., 2003).

In discussing the maintenance of cellular connectivity, I proposed above that cell division maintains identity or phenotype by regenerating the original patterns of connectivity. This can now be seen to be an over-simplification. More exactly, division is fundamental to generating the two classes of patterns of connectivity that allow a population to live on the scales. In other words, even in steady-state conditions, growth, and division create a bacterial population with a constant distribution of varied but coherent phenotypes that interchange over successive generations.

## Intensity Sensing and Quantity Sensing

The cell cycle is also the solution to another two fundamental problems. The *intensity* problem that confronts bacteria during growth is that the **NE** constituents that do the work, like enzymes, eventually work with such intensity that they can do no more; the cell can then do no better than grow linearly. The *quantity* problem that also confronts bacteria during growth is that the quantity of unused, **E** material – be it lipids or macromolecules – accumulates; this material risks being a waste of resources. By sensing both intensity and quantity, a bacterium could decide when it was time to increase the number of enzymes and to convert the unused macromolecules into another form. In the case of *E. coli*, we have proposed that this combination of intensity and quantity sensing reaches a threshold that triggers the cell cycle and it is then the events of the cell cycle that solve both intensity and quantity problems (Norris, 2011; Norris and Amar, 2012a; Norris et al., 2014). The idea is that the state of one or more **NE** hyperstructures reflects the intensity of metabolism at that *particular moment in time* and that when this intensity is sufficiently high for growth to risk growth being limited, one or more signals are emitted by these hyperstructures to initiate chromosome replication. In addition, the state of one or more **E** hyperstructures senses the quantity of accumulated material and when this is likely to be sufficient for daughter cells to be generated, a complementary, initiation signal is emitted. These signals trigger the cell cycle which has the function of converting **E** into **NE** material and redistributing this material into the daughter cells.

There are several mechanisms that might be responsible for intensity sensing; one attractive mechanism could be based on interactions between cytoskeletal hyperstructures and metabolic enzymes such that, for example, an enzyme that is catalyzing its reaction has a greater affinity for the replication and/or cytoskeletal hyperstructures and helps to stabilize/destabilize them (Norris et al., 2013). This actually occurs in *Bacillus subtilis* where assembly of FtsZ into an effective division structure is inhibited by interaction between FtsZ and the glucosyltransferase UgtP, which depends on the concentration of UDP-glucose (Weart et al., 2007); a similar system exists in *E. coli* where the interaction is between FtsZ and the functional homolog of UgtP, OpgH (Hill et al., 2013). FtsZ dynamics are also affected by the location of pyruvate dehydrogenase E1α, which depends on the concentration of pyruvate (Monahan et al., 2014). Another mechanism might involve sensing the density of RNA polymerases transcribing genes and of ribosomes translating mRNA (Norris, 1995c); in line with this, a correlation exists between attaining a particular growth rate per unit mass in mouse lymphoblasts and their entry into S phase (Son et al., 2012). There are also several mechanisms that might be responsible for quantity sensing and that, for example, might be based on the possible accumulation of proteins and lipids (Norris and Amar, 2012a). In the former case, examples might include the wall synthesis enzyme, MurG, which is stored in an inactive form in the cell poles (Michaelis and Gitai, 2010) and the CTP synthetase, CtpS, which is stored in an inactive form as a polymer (Barry et al., 2014). In the latter case, accumulation of cardiolipin would be an attractive candidate given its roles in initiation of replication (Sekimizu and Kornberg, 1988; Makise et al., 2002) and in cell division (Mileykovskaya and Dowhan, 2000; Koppelman et al., 2001; Kawai et al., 2004). Finally, there are molecules of major physiological importance that are often overlooked (Norris et al., 2015); these include polyphosphate which, in some bacteria, has been shown to play a role in the cell cycle (Boutte et al., 2012) and to exist in both soluble and insoluble forms (Klauth et al., 2006).

## Competitive Coherence

*Competitive coherence* is a particular way of choosing a temporal series of subsets of elements from a much larger set (Norris et al., 2012). These subsets are *active* subsets insofar as a subset actively determines the behavior of the entire system at a particular time. Such a subset (*alias* the phenotype) is selected by a competition between (1) those elements that have a relationship with the elements in the previous subset and (2) those elements that have a relationship with the elements already selected to be part of the new, emerging subset. At the level of a bacterium, growth and survival require selection of an active subset of macromolecules in response to external and internal conditions; such responses entail both the generation of a coherent cell state, in which the cell’s contents work together efficiently and harmoniously, and the generation of a coherent sequence of cell states. Incoherence within a cell state is punished since, for example, a cell that simultaneously induces the expression of genes for growth at high temperature and at low temperature is likely to be outcompeted by rival cells that induce each set of genes only when needed. Incoherence in the succession of cell states is also punished since a cell that goes from one cell state to another very different one (without good environmental reason) is wasting resources. A strong selective pressure, therefore, exists to generate active subsets of elements to provide both coherent cell states and a coherent sequence of such states (Norris et al., 2014). In response to this pressure, the process of competitive coherence has evolved to reconcile two sorts of coherence in generating the active subset. Note that the active subset is not the same as the bacterium’s entire set of genes, set of mRNAs and/or set of proteins: the active subset is the set of elements that determines the behavior of the cell at a particular time. Hence, a gene that is not expressed or an mRNA that is not translated or an enzyme that is not in the right hyperstructure might have no effect on the phenotype at a particular time and therefore would not be part of the active subset. The question therefore arises as to whether the concept of competitive coherence can provide an insight into the cell cycle.

One of the important parameters in competitive coherence is the nature and size of the active subset. It is far from clear, however, how the size of the active subset should vary with conditions. In a bacterium growing rapidly, the need for it to be ready to adapt to new opportunities or stresses might be better served by a large active subset containing mainly **NE** elements whilst in a non-growing bacterium the need for it to maintain its structures (rather than abandon them too readily so as to profit from a growth opportunity) may require a small active subset containing mainly **E** elements. It might be argued that in stress conditions, the active subset may be smaller in order to conserve energy and to lose diversity as the system concentrates on a limited number of **E** hyperstructures in which the elements are tightly connected with one another (Norris et al., 2014). In changing conditions, however, we predict that the active subset would also change and go through a period when it changes in size, as proposed for *Streptomyces coelicolor* as it goes through changes in nutrient availability and population density (Vohradsky and Ramsden, 2001).

Changes in the active subset is where the cell cycle comes in. Cell division permits activity subsets not just with different contents but also of different sizes. One question here is what would happen to the active subset if a bacterium were to grow without going through the cell cycle. Presumably, RNA and protein production would eventually become limited by the relative scarcity of DNA so that exponential growth could not be sustained (Zaritsky, 1975; Norris, 2011). It is tempting to think that, since many different genes compete for an increasing number of RNA polymerases (Stickle et al., 1994), continuation of growth in the absence of a cell cycle would lead to the inappropriate transcription of an increasing number of genes and to an incoherent phenotype, but this must depend to some extent on how the regulatory network would change. Assuming an increase in transcriptional and translational machinery relative to unit DNA, growth without cell cycle progress should lead to a gene encoding a repressor reaching a maximum level of expression and the repressor eventually being diluted by growth and becoming insufficient to repress its targets. It is therefore conceivable that the absence of the cell cycle would result in an increase in the size of the active subset such that all the phenotypes in the population converged onto a single phenotype. Inhibition of chromosome replication in *B. subtilis*, accompanied by continued growth led to significant alterations in the expression of over a 100 genes, around a half being regulated by DnaA, which is both an initiator of replication and a transcription factor (Goranov et al., 2005). In this experiment, the period of unbalanced growth lasted for a maximum of 90 min and only the mRNA levels of the entire population could be measured. To explore the ideas outlined above would require following the global patterns of transcription and translation in individual cells growing over several hours without cell cycle progress. Unfortunately, this is at present impossible. In summary, once again, the cell cycle is about the preservation of the coherence of individual phenotypes and the coherence of the population of phenotypes.

## Discussion

It is somewhat surprising that after decades of intense research into the cell cycle of some of biology’s best understood model organisms, the fundamental nature of the control over the cell cycle remains elusive and, in particular, the coupling between the cell cycle and the growth rate (Marshall et al., 2012; Campos et al., 2014; Taheri-Araghi et al., 2015). One explanation is that the paradigm that has guided this research is wrong. If so, a new paradigm is required. This is easier said than done. The approach to generating new paradigms that I advocate here is to ask the question “why do bacteria divide?" Such ‘existentialist’ questions are fundamental and attempts to answer them can lead to new insights. Some of us have considered explanations for cell division in terms of the maintenance of cellular connectivity, the reconciliation of growth and survival strategies, and combining present coherence with past coherence. Could these ideas lead to a new paradigm and, if so, would we know if it were a good one?

A new paradigm might be evaluated on grounds that include its esthetic qualities, its testability, its difference with the current paradigm, and the breadth of its fields of application (Norris, 1995a). In other words, does the new paradigm result in a rapid advance in a specific field of science, and does it help us understand other fields? One approach is to focus on those *unifying* problems that recur in different fields, for example, how to limit the enormity of phenotype space so that Darwinian selection can act (Kauffman, 1996). To solve this problem, we have invoked a key role for simple, universal molecules and inorganic ions, termed SUMIs, which include polyphosphate, polyhydroxybutyrate, polyamines, and calcium (Norris et al., 2015). A complementary approach is to ask whether the paradigm would help elucidate another field, for example, could a hypothesis developed for the origins of life help with cell division? This may be the case for the SUMIs, all of which have major roles in the growth, cell cycle, and differentiation of bacteria (Huang and Reusch, 1996; Igarashi and Kashiwagi, 2006; Naseem et al., 2009; Rao et al., 2009; Boutte et al., 2012). It may also be that case for **NE** and **E** hyperstructures, dualism, and life on the scales, which we would argue are as important to the division of modern cells as they were to the origins of protocells in the prebiotic ecology (Norris and Raine, 1998; Hunding et al., 2006; Norris et al., 2007b). Indeed, concepts such as life on the scales and competitive coherence are essentially scale-free and substance-free, and are applicable to life elsewhere in the cosmos (Norris, 2015).

In discussing the maintenance of cellular connectivity, we proposed that cell division maintains identity by regenerating the original patterns of connectivity. This is clearly an over-simplification since division is fundamental to generating the two patterns of connectivity that allow a population to live on the scales. The point is that growth and division create a population of individuals with a constant distribution of phenotypes or identities that interchange over successive generations. Ironically, this fundamental role for the cell cycle may have been obscured by the importance – admittedly understandable – attributed to only studying the cell cycle of bacteria growing in steady-state conditions which minimize differentiation (Schaechter et al., 1958; Fishov et al., 1995).

## Conflict of Interest Statement

The author declares that the research was conducted in the absence of any commercial or financial relationships that could be construed as a potential conflict of interest.
